# Do your own research!

**DOI:** 10.1007/s11229-022-03793-w

**Published:** 2022-08-20

**Authors:** Neil Levy

**Affiliations:** 1 Department of Philosophy, Macquarie University, Sydney, NSW 2109 Australia; 2 Uehiro Centre for Practical Ethics, University of Oxford, Oxford, OX1 1PT UK

**Keywords:** Knowledge, Understanding, Conspiracy theories, Intellectual autonomy

## Abstract

Philosophical tradition and conspiracy theorists converge in suggesting that ordinary people ought to do their own research, rather than accept the word of others. In this paper, I argue that it’s no accident that conspiracy theorists value lay research on expert topics: such research is likely to undermine knowledge, via its effects on truth and justification. Accepting expert testimony is a far more reliable route to truth. Nevertheless, lay research has a range of benefits; in particular, it is likely to lead to greater understanding, even when it does not lead to knowledge. I argue that we can reap most of the genuine benefits of lay research while minimizing the risks by engaging in exploratory, rather than truth-directed, inquiry. To engage in exploratory inquiry is to engage dogmatically, expecting to be unable to confirm the expert view or to disconfirm rivals.

Q: Why did the conspiracy theorist cross the road?

A: Do your own research!

The joke may be weak, but it points to an interesting challenge. Contrarian thinkers—including those people disparaged as ‘conspiracy theorists’—are often proud of their intellectual autonomy. ‘Do your own research’; ‘think for yourself’; the accusation that other people are mere ‘sheeple,’ blindly accepting everything fed to them by government and mainstream media—these familiar slogans and phrases express the heavy emphasis they place on thinking for themselves. Contrary to almost equally familiar tropes, moreover, these contrarian thinkers often have good grounds for their pride in their intellectual autonomy.

The utility and descriptive accuracy of the term ‘conspiracy theorist’ is highly contested (Coady, 2003; Dentith, 2014; Pigden, 2017). Since these controversies are orthogonal to the issues I’m concerned with here, I avoid using it here. I’m concerned not just with those theories that are described as conspiracy theories, but with *contrarian theories* more generally: theories that conflict with what Coady (2003) calls the ‘official story’. More narrowly still, I’m concerned with theories that conflict with the official stories of *epistemic authorities* (that is, those people socially acknowledged as the relevant experts on a topic). Such theories are, of course, common. Well-known examples include theories about the moon landing, the QAnon conspiracy theory and the belief that the vaccines against Covid-19 kill more people than the disease.

Those who accept contrarian theories often advocate doing your own research to test the official stories. Conversely, those who accept the official stories and denigrate contrarian theorists often accuse them of credulousness in believing what they hear or read (paradigmatically, on social media), and take doing our own research as the *antidote* to such theorising; they disparage contrarian theorists as all talk when it comes to doing their own research.

The available evidence suggests that, contra the stereotype, contrarian theorists are actually quite likely to do their own research; that is, to engage with and attempt to assess the first-order evidence. If those who are active on the conspiracy subreddit—probably the largest and most influential online discussion forum for conspiracy theories (Klein et al., 2019)—are representative, contrarian theories are often developed and elaborated by people who are very concerned with gathering and assessing evidence. They spend a significant amount of time reflecting on the status of various sources of information *as* evidence, and pride themselves on being discerning in what conspiracies they accept (Klein et al., 2018). Similarly, work on online groups promoting dissent from the official story on masks and other measures as a response to Covid-19 finds that these groups prize grappling with the data. Some ban links to interpretations of data from outside the group. Instead, they encourage members to analyse the raw figures (e.g. county-by-county data on mask usage and infection rates) for themselves, and they even hold tutorials on how to gather and analyze data (Lee et al., 2021). This empirical work backs up the intuition and observations of some philosophers, that contrarian theorists are often *more* engaged in the search for and the evaluation of evidence than are most people who accept the official story (Harris, 2018).

In this paper, I will argue that it’s no accident that these theorists laud doing their own research: rather, doing one’s own research, in the manner they (and philosophical tradition) envisage, is the royal road to contrarian theorizing. On topics on which there is a large body of truly expert knowledge, and in which there are official stories (or a range of official stories: a range, that is, of genuinely expert views), doing one’s own research is epistemically risky. Compared to accepting the testimony of experts, thinking for oneself risks knowledge in two ways: by making it more likely we will end up believing falsehoods and by threatening to undermine justification. In light of this fact, we must reassess the value of an activity that philosophers have lauded for at least three centuries. I will suggest that thinking for ourselves—engaging with first-order evidence—remains valuable, but that it is best undertaken in the service of understanding, rather than knowledge. We retain knowledge and gain understanding best, I will argue, when we engage with the first-order evidence dogmatically: placing little weight on the evidence such claims may seem to provide against the expert view(s).

## The value of engagement

Thinking for oneself has long been lauded by philosophers. Kant (1784/1991) calls on us to emerge from “immaturity”, which he characterizes as “the inability to use one’s own understanding without the guidance of others.” Locke (1689/1975) warned “we may as rationally hope to see with other Men’s Eyes, as to know by other Men’s Understandings.” Descartes (1701/1985) set down as one of his *Rules for the Direction of the Mind* that we rely only on “what we can clearly and evidently intuit or deduce with certainty, and not what other people have thought….[f]or knowledge can be attained in no other way”. All preach the centrality of epistemic self-reliance.

Of course, between their time and ours, epistemology has been transformed in multiple ways.^1^ Most relevantly for our purposes, we’ve come to a much greater appreciation of the centrality of social processes to the generation and transmission of knowledge. Correlatively, we’ve redefined intellectual autonomy to allow for the acquisition of knowledge via testimony.^2^ Testimony provides higher-order evidence, and we now all recognize that agents can acquire knowledge on the basis of such evidence. However, we haven’t ceased thinking that it is better, and often necessary, to engage directly with the first-order evidence in roughly the manner urged by the philosophical tradition.

Reasons for valuing engagement with first-order evidence are various. On some views, there are kinds of knowledge (moral knowledge or aesthetic knowledge, for instance) that can’t be acquired through testimony (Hills, 2009; McGrath, 2009). On other (compatible) views, *understanding* can’t be acquired via testimony (Pritchard, 2016; Zagzebski, 2007; see Boyd, 2017 for dissent). But there appears to be something close to a consensus that intellectual self-reliance is valuable. Even if I *can* gain knowledge, or even understanding, by testimony, it is better to see things for myself. Most people accept, of course, that engagement like this is just one good among many. Nevertheless, *ceteris paribus* it is better to acquire knowledge on our own, many epistemologists think.^3^

In ‘doing her own research’, then, the contrarian theorist appears to engage in a kind of intellectual activity we value. But it’s an open question whether we should value it as much as we do. We often and predictably do much better *with regard to knowledge* by relying on testimony, rather than by doing our own research. The former is routinely more reliable than the latter; doing our own research risks the truth of our beliefs and their justification (Matheson, 2022).

In doing her own research, the contrarian theorist runs a risk: coming to a false belief is a predictable result. There is, after all, a great deal of misleading but apparently plausible information available to her. She is (typically) a layperson, when it comes to the topic she’s considering (why the Twin Towers collapsed; the origins of Covid-19; the election of Donald Trump). She has no special expertise when it comes to evaluating claims about the melting temperature of steel, the genetic sequencing of a virus or the influence of Twitter bots on people’s choices. She engages with the first-order evidence as a layperson must: by reading competing accounts and rebuttals, and evaluating them. In the absence of genuine expertise, however, these are immensely difficult tasks, and it’s unsurprising if diligent inquiry leads her astray.

Epistemologists often underestimate the difficulty of assessing the actual import of the first-order evidence. Cassam (2018), for example, regards a *failure* to engage in this kind of task as epistemically vicious. Elsewhere, I’ve argued that Cassam’s arguments seriously underestimate the degree and the specificity of the expertise it takes to responsibly assess claims; even expertise in closely related fields may leave the person ill-equipped (Levy, 2021a, b). It’s worth emphasising just how field-specific expertise is (Kilov, 2020): possessing expert-level knowledge in cognitive neuroscience, say, will often leave someone unable competently to assess the claims of a developmental neuroscientist; being an expert in Renaissance Italy may leave someone unable to assess claims made about the baroque (indeed, the specificity of expertise is greater than that suggests: the Renaissance historian may know a great deal about agriculture and markets, and little about the intellectual life of the cities. When it comes to evaluating claims about *that*, she may be unable competently to assess competing claims).^4^

If all this is true, then laypeople regularly do better to defer to testimony than to inquire for themselves, at least so far as the aim is acquiring knowledge. On every question that requires expertise to answer and on which there is an expert consensus (or on which expertise is required for a reasonable view), deference can be expected to do better than self-reliance. Doing your own research on a topic where an agent lacks the expertise to distinguish trustworthy from untrustworthy sources or to properly interpret and weigh different kinds of evidence is an unreliable way of forming beliefs. Even if it leads to true belief, there are good reasons for both externalists and internalists to doubt that it leads to knowledge: the process is unreliable, insofar as it can easily go astray, and the agent is liable to end up possessing undefeated defeaters (the mass of competing claims they can’t address) for their beliefs. Deferring to the expert consensus, on the other hand, is a much more reliable way of acquiring knowledge: at worst, it typically does no worse than doing your own research and at best it does much better.^5^ So autonomous enquiry has little to recommend it on that score.

If doing your own research is epistemically risky, and deference a surer route to knowledge, should we cease to engage in it on our own behalf, and alter educational policy as well as our personal policy of offering advice to discourage it? That is wildly counterintuitive. Fortunately, there’s an alternative. Before I discuss it, however, it’s important to address some obvious objections. In doing so, I make some concessions: there are circumstances in which doing one’s own research is the best route to knowledge. But these concessions are limited: these cases are rarer than they appear.

## Engaging for knowledge

Experts can and must do their own research. In some fields, engagement in primary research on a topic is a necessary condition of counting as an expert on it. In others, one may count as an expert even if one doesn’t do primary research on that topic, but even in those fields, of course, expertise exists in virtue of some experts being engaged in primary research. Worries about doing one’s own research don’t apply to the expert, at least to anything like the same extent: in their own field, their expertise ensures that the epistemic risks are much lower for them than for us, and in any case their research is indispensable to the progress of knowledge. Experts run much the same epistemic risks as laypeople when (as happens too often) they overestimate the breadth of their expertise, but in their own narrow field they are competent to assess competing claims. Beyond these cases, situations in which people doing one’s own research is conducive to knowledge are few.

One kind of case involves those who realistically aim to acquire genuine expertise. It should be stressed that on most topics, most of us have little prospect of acquiring genuine expertise. We may lack the capacity to acquire the requisite maths, or requisite linguistic competence or whatever other specialist tools we’d need. We may simply lack the time and other resources or the considerable commitment needed for the acquisition of these skills. All of us must make intellectual choices, deciding where to devote our finite intellectual resources. None of us can acquire genuine expertise across the full range of topics we’d like to know about. Few of us can reasonably engage in our own research in view of the prospect of acquiring expertise on more than a small handful of topics across our lifetimes.

Even those who realistically aim at expertise must wait until they are well along the road to it before research can confidently be advised for them. At earlier stages in our intellectual itinerary, we lack a good grasp of what it is important to understand and in what order we must grapple with material. Research without close guidance may never result in the acquisition of full competence (think of Kuhn (1970) on how the education of scientists requires that the trainee be brought to see her discipline through the reigning paradigm, by way of a highly selective, and somewhat misleading, presentation of the history of her field and the state of the art). Further, if one engages in research prior to the acquisition of expertise, one risks acquiring false beliefs which may subsequently become entrenched. If we are responsibly to engage in our own research, we do better to wait until we have acquired some substantial degree of genuine expertise, rather than enter into it before we have the tools to weigh evidence appropriately.

There is, however, a *kind* of research in which non-experts must sometimes engage. We are required to engage in what I will call *shallow* research. Shallow research consists in the consultation of sources we have good reason to regard as reliable and which are aimed at non-experts like us. We engage in shallow research by reading mainstream media, trade books and the like, attending public lectures and so on. We are required to engage in shallow research when we encounter competing views, and are unsure which to accept. Consider Cassam’s (2018) principle example of what he sees as responsible inquiry into a contrarian theory: responding to David Irving’s Holocaust denial. Cassam argues that to refute Irving we must do some research: for instance, read a trade book by the historian Richard Evans and the Wikipedia article on Irving. Following Cassam’s sensible advice is engaging in shallow research. But shallow research is not doing one’s own research, as philosophical tradition and contrarian theorists conceive of it.

Shallow research is not an *alternative* to deference: it is aimed at and guided by deference. The goal of the person who engages in it is to assess to whom she should defer. Engaging in shallow research doesn’t involve trawling the Bundesarchiv in Berlin for evidence that supports or undermines Irving’s claims, or even in a careful comparison of the voluminous secondary literature on the Holocaust. It involves a search for evidence about whether we should defer to Evans and Wikipedia, on the one hand, or Irving, on the other (who has better credentials or is supported by an expert consensus?) Shallow research is also *guided* by deference. In assessing who we should defer to, we *assume* the reliability of the mainstream sources (that Wikipedia and books like the one by Evans accurately report, say, Irving’s errors as a historian). The person who reads Evans to assess Irving’s plausibility does not *merely* defer. She also assesses the plausibility of Evans’ arguments against Irving. But she does so in a way that remains guided by deference: she assumes the broad reliability of Evans’ factual claims and is disposed to weigh his arguments more heavily than Irving’s, since she takes him to speak to with the weight of the consensus.

Some degree of shallow research—the consultation of further, very mainstream, sources—is a normal and virtuous part of the acquisition of knowledge by responsible individuals. To the extent there’s a consensus on a topic and the media is responsible in reporting it, such research is a reliable route to knowledge. In an appropriately structured epistemic environment, such research remains responsible even in the absence of an expert consensus. In such an appropriately structured environment, the public sources should report the range of reasonable views, and the risks of shallow research to knowledge are low. Of course, the epistemic environment is often not structured appropriately, as the example of climate change illustrates. In these cases, shallow research may risk knowledge (reading the op-ed pages of the *Wall Street Journal* may lead someone with true beliefs on climate change to come to reject those beliefs, or undermine her justification for them). But that fact offers no support to those who commend deeper research. There’s no reason to expect deeper research to do any better in this hostile epistemic environment.

Shallow research is the only kind of research most of us are called upon to engage in with regard to the significant truths about matters within the purview of epistemic authorities. It’s not an alternative to deference: it is aimed at and guided by deference. Of course, such deference is never a guarantee of knowledge. Conspiracies *do* occur and error sometimes ramifies; deference can fail to secure knowledge for these reasons. But even under these conditions, doing our own research rarely outperforms deference. Even if genuine epistemic authorities are rare (something I do not believe), deference has a higher expected epistemic return than self-reliance. Self-reliance does not tend to outperform unreliable authority. It can be expected to do worse than reliable authority and no better than unreliable authority.^6^

## Engaging for understanding

For the person who lacks a substantial degree of expertise, doing anything more than shallow research comes with considerable risk to knowledge. So much the worse for doing our own (substantive) research? In this section, I will argue that deeper research may responsibly be pursued, with the aim of coming to *understand*, rather than to know.

The nature of understanding is subject to ongoing dispute.^7^ Like many other writers on the topic, I won’t attempt to define it in any precise way, let alone provide an account. I’ll content myself with a few reminders. Understanding is manifested in an ability to answer *why* questions, and therefore depends on a grasp of causes and connections. Someone can know *that* something is the case without knowing *why*: many people who accept the reality of climate change (for instance) are in this boat. Understanding comes in degrees, of course, and many of us have *some* understanding of climate change. We know that climate change is caused by CO_2_. We can give a broad answer to an important ‘why’ question and take a stab at assessing some counterfactuals (what would happen if we released less CO_2_?) Laypeople differ in how much understanding they have, but they lack (more or less by definition) broad and deep understanding. They grasp fewer causal connections, they can answer fewer and vaguer ‘why’ questions and assess fewer counterfactuals (what would happen if we converted electricity production from coal to natural gas?).

We’ve seen that deep research, the substantive research that both contrarian theorists and philosophical tradition lauds, risks knowledge, either by threatening truth or by threatening justification. The good news is that the risks with regard to understanding are much smaller. Understanding is widely held to be more valuable than knowledge; perhaps surprisingly, in that light, it is also less vulnerable. How much less vulnerable understanding is than knowledge is controversial. As Grimm (2017) notes, there is a widespread sense that understanding is, entirely or to a much greater extent than knowledge, an internal states of agents. If that’s the case, then agents may be incorrigible (or at least highly reliable) with regard to whether they possess it. Correlatively, understanding might be insulated against the external factors that prevent beliefs from failing to qualify as knowledge.

On some views, understanding is insulated from the factors that prevent justified true beliefs from amounting to knowledge (Hills, 2015; Pritchard, 2009). Suppose malicious pranksters subtly scramble every article about climate science on a website to make them incoherent. By mistake, they subject one article to the scrambling procedure twice, and the effects of the second scramble cancel out the effects of the first. Many epistemologists would deny that Axel acquires knowledge from that article, but accept that he acquires understanding from it. He can, after all, correctly answer *why* questions and assess counterfactuals correctly (see Hannon, 2021 for discussion).

Some philosophers argue that understanding can persist not only in the face of knowledge-undermining luck, but also in the absence of justification. On some views, an agent may understand something, but fail to believe their own explanatory account of it (Wilkenfeld, 2017). Such an agent may possess understanding without justification, in virtue of their possession of an undefeated defeater for their explanation (Hills, 2015). It is controversial whether internally accessible defeaters, that undermine knowledge, may nevertheless be compatible with understanding (Dellsén, 2017; Hannon, 2021). But it is not controversial that someone may retain understanding in the face of internally *in*accessible defeaters *whether or not* they undermine knowledge. That is, though it is controversial whether (or when) epistemic luck undermines knowledge, philosophers on both sides of this debate accept that understanding survives the threat. Agents who acquire true beliefs through their own research may sometimes lack knowledge, for example because their beliefs were acquired from the few facts on an otherwise unreliable website, or because they read the one reliable site among the dozens they had bookmarked. Nevertheless, insofar as they can correctly answer *why* questions, assess counterfactuals and so on, they genuinely have understanding.

Someone may even come to embrace a wild conspiracy about an event (say) while nevertheless genuinely increasing her understanding of central aspects of it. Consider the person who investigates the collapse of the World Trade Center and in doing so comes to accept a conspiracy theory about its causes. She believes that the official sources were complicit in the attack and that the official narrative has been doctored to hide this fact. She is therefore sceptical about the timeline and sequence of events reported; on any account of ‘knowledge’, she does not know these basic facts. Nevertheless, she might come to understand the official narrative very much better than most of us: how the pieces fit so conveniently well (as she sees it) together, where the planes (supposedly) were and when, and how the collapse would have occurred had the story been true.^8^ She may be able to answer many *why* questions we (who have not done our own research) can’t (why would *those* flights have been selected?). She might even be able to assess counterfactuals we cannot (what would have happened if the plane had impacted at a different angle?) Her understanding has genuinely increased through her research.^9^

Of course, understanding *can* be lost as a result of deep research. We might become confused by the competing and conflicting accounts we encounter. This is a real risk, of course, but it is usually diagnosable from within and can be remedied through more research (generally speaking, if further research cannot remedy it—perhaps because it requires intellectual tools we can’t reasonably expect to acquire, or the state of knowledge is too incomplete—than we were wrong in thinking we understood prior to our research, so our confusion reflects an *improvement* in our epistemic situation). There’s also a risk that doing our own research might lead to an inflated sense of our understanding. The sense of fluency that arises from familiarity with a topic might contribute to the *illusion of explanatory depth* (Mills & Keil, 2004; Rozenblit & Keil, 2002): our tendency to think we understand something merely because it is familiar. Once again, however, the risk will be small for most topics, not because the effect won’t arise but because those who don’t do their own research tend to be subject to it anyway: ordinary cultural familiarity with 9/11 or Covid-19 might be enough to lead to the illusion in most of us. Further research might lead to a greater sense of familiarity, but there seems to be no a priori reason why the *gap* between perceived understanding and actual understanding should tend to grow.

Doing one’s own research is therefore valuable because it can lead to understanding. It is an unreliable and risky route to knowledge, but may be indispensable for epistemic outcomes that are also very valuable. There’s therefore a prima facie case for doing one’s own research, not *instead of* deferring, but alongside it. Jonathan Matheson (Matheson, 2022), who has identified the conflict between doing one’s own research and securing knowledge before me, prescribes a similar response: research for understanding; deference for truth. But Matheson overlooks the risks of doing one’s own research. How are we to hang on to knowledge if our research seems to fail to confirm the official story? I’ll suggest that such failures are common. And of course contrarian theorists are keen to bombard us with evidence that, often, really seems inconsistent with the official story. Isn’t the risk of losing knowledge too great to justify the expected gains in understanding?

We seem to confront a dilemma. If we engage in our own research, we may secure understanding but we risk knowledge. If we simply defer, we lose understanding. We also risk other benefits. After all, sometimes the official story is false, and lay research helps uncover this fact. As Coady and Pigden stress, conspiracies are sometimes real (Coady, 2012; Pigden, 2017), and occasionally digging by laypeople uncovers them. These kinds of cases occur when those in powerful positions are able to create a consensus (via manipulation of information or by suborning epistemic authorities), or to create a sufficiently convincing appearance of a consensus by strategic promotion and discrediting of experts (the lead up to the Iraq war, with an apparent expert consensus around WMDs, may be an example of the latter). In these kinds of cases, non-experts may have certain advantages over experts in uncovering the truth. Experts may be hampered by their dependency on the conspirators. Some experts, for example, depend on ongoing access to official sources for their work, and will be unwilling to go out on a limb, on a mere hunch, if that threatens their access. Others will be mindful of their reputations and how they can be smeared with the accusation ‘conspiracy theorist’. Moreover, when evidence has been hidden or obscured, a non-expert might just happen to be in the right place to detect it; whistleblowers play a valuable social role.

The (very unlikely, but very valuable) possibility of uncovering a conspiracy or bringing to light new evidence is one epistemic benefit of lay research. There are others. Lay discoveries are rare but real, and their occurrence also speaks in favor of lay research. Important contributions by non-experts are especially likely when the expert consensus excludes certain voices (women or indigenous people, for example) but touches on their concerns. For instance, while not counting as experts (at least not by the criteria the experts use), indigenous people may be able to make indispensable contributions with regard to land management or the medicinal value of plants. In other cases, non-experts may make important contributions without making any true claims. A non-expert might be able to pose a challenge to the consensus view to which the genuine experts are unable to respond immediately, but which motivates them to extend their theory in productive ways. Both evolutionary theory and the science of climate science plausibly benefitted from ill-motivated attacks by non-experts, which nevertheless identified gaps or obscurities and led to their filling (see Dennett, 1996 for examples from the history of evolutionary theory).

There are also sometimes important social benefits to doing one’s own research. For example, the acquisition of genuine understanding, and the capacity to speak in the technical vocabulary of a discipline, may be necessary for marginalized voices to be heard and given due weight. One famous example involves AIDS activists, who discovered they needed to be able to use the language of virology to have their voices heard (Epstein, 1995). By acquiring genuine competence with the relevant science, they were able to exercise genuine influence, and make research more inclusive of the whole population of sufferers.

While discouraging people from doing their own research will tend on average to protect the knowledge of lay people and slow the generation and promulgation of unwarranted, and sometimes dangerous, conspiracy theories, these are significant costs, both to individuals themselves and to the broader community. Matheson may be right that we need to combine research with deference, but how, exactly, are we to do this, such that we maximize the benefits and minimize the risks? I doubt there is any risk-free way to pursue one’s own research. But there is a way to minimize the risks without eliminating the benefits. We ought to encourage *exploratory* inquiry, aimed at understanding, rather than the *truth-directed* inquiry that is the royal road to the loss of knowledge.

## Exploratory and truth-directed inquiry

Our paradigm of inquiry is truth directed: we inquire in order to find out. An agent engages in truth-directed inquiry into an event, *e* (for example), either because it is unknown (to her) how *e* came about, or because she doesn’t fully trust the received view about *e*. Scientists, historians and detectives, at least in the popular imagination, conform to this model. The scientist might investigate *whether Hurricane Sandy was caused by climate change* or the historian *whether the rise of fascism was attributable to the Versailles Treaty*: there is some question they regard as important (or interesting) and they have good reason to believe that inquiry is the most reliable way to find out. Often, the investigator is not the first person to inquire into the question, but further or fresh inquiry is usually legitimate. A well-worn movie plot involves the detective who reopens a case long believed solved, because they have a niggling suspicion that wrong person was convicted. Here, too, the inquiry is truth-directed: undertaken in order to find out.

But inquiry isn’t always truth-directed. We may investigate despite being more confident that we know the truth about the event or the process than we are that our inquiry is able to confirm its causes. Think of high school science classes. Students may be given the task of performing an experiment that is supposed to demonstrate some well-established law. Notoriously, these experiments often fail to yield the results they’re supposed to. Students should not, and their teachers do not, conclude that Hooke’s law (or whatever it is) has been falsified. They think that experimenter error is a far more likely explanation than a local deviation of the universe from its usual course or a mistake having been propagated down the centuries. The fact that they so easily set aside the actual results of the experiment indicates that they did not undertake it *in order to find out*. Instead, it was aimed at understanding and not truth.^10^

An agent engages in exploratory inquiry when she is not particularly concerned about the results of the (token) inquiry. She may be unconcerned because she takes herself already to know the results (or what the results would be, were she to carry it out carefully enough), or because she believes that the results are already recorded in textbooks or journals, or simply because she doesn’t care about the result. A scientist might perform a novel experiment with new apparatus in order to acquaint herself with the apparatus, without being concerned what results they yield.

When contrarian theorists urge us to “do our own research”, it is of course truth-directed inquiry they have in mind. They advocate not accepting the official story (about 9/11, vaccines, climate change) on trust, but instead finding out for ourselves. They are passionately concerned with the results of the inquiry, not the process (as we saw, they may be attentive to the process, but they attend to it in order to make the results more reliable). Descartes, Locke and Kant, too, are concerned with truth-directed inquiry. They are motivated by the conviction that only when we have confirmed findings for ourselves will our knowledge be secure.

It's truth-directed inquiry that is most risky. We risk knowledge in undertaking it. We will be lucky if we hit upon or retain true beliefs through truth directed inquiry, and luckier still if those beliefs are well enough justified to count as knowledge. Of course, contrarian theorists and the canonical philosophers who urge its importance are right in thinking that it’s important: truth-directed inquiry is essential to the progress of knowledge. But truth-directed inquiry by laypeople, on topics that have been subjected to a great deal of scrutiny by diverse experts, has epistemic costs that routinely far outweigh its benefits.

Exploratory inquiry is much less risky, and we can undertake it ourselves (and advocate it for others) in much better conscience. I can undertake inquiry into climate change or vaccines in much the same spirit as the high school student performing a classroom experiment on Hooke’s law. Like her, I can have an incomparably higher degree of confidence in what the results of inquiry *should* be than in its actual results. Like her, I can take any divergence between what I seem to find out and the consensus view as a reason for suspicion about my inquiry rather than a reason to doubt the consensus. Like her, I undertake the inquiry to better understand, rather than to find out.

Above, we saw the lay inquiry had a number of advantages. One such benefit is epistemic: lay inquiry sometimes corrects the expert consensus. This may occur when certain voices are inappropriately excluded from the conversation (due to prejudice, for example), when a consensus forms prematurely or when evidence is suppressed. All these worries seem to call for truth-directed inquiry: it is only if we put significant weight on any divergence between the consensus and the results of lay inquiry that we can correct the former, and this requires a concern with the results of inquiry. If engaging in exploratory inquiry entails putting a very heavy weight on the official story, such that *any* divergence between what seems to be supported by the inquiry and the official story is taken as evidence that the inquiry has failed, then we cannot reap the epistemic benefits. But exploratory inquiry doesn’t require that we weigh the official story infinitely more heavily than the results of inquiry.

If inquiry is to be genuinely exploratory, we must place a very heavy weight on the official story compared to the results of our inquiry. We must indeed think that divergence is much better evidence for the hypothesis that our inquiry was in some way defective than for the hypothesis that the official story is wrong. We should take failure to confirm the official story (whether by direct experiment, as in a high-school science class, or by reading articles on the internet) as a reason to try again, and we should regard repeated failures as strong evidence that we lack the skills to adjudicate the question. Just how much greater weight we should place on the official story than on our own inquiries is a difficult question. I doubt we can quantify the difference in weight, beyond saying that it is large. Plausibly, too, the difference should be greater with regard to some questions than others: some are entirely beyond the competence of most of us (string theory), or require special equipment and large teams of experts, whereas others are somewhat more tractable to lay investigation. On the most specialized questions, the weight we should place on the official story is perhaps heavy enough to prevent us *ever* from being justified in rejecting it on the basis of exploratory inquiry: failure to confirm it is always more likely to be due to our limitations than its falsity.^11^

But on many questions we *can* produce evidence against the official story through exploratory inquiry. Inquiry is exploratory only if we’re disposed to take our failure to confirm the official story as evidence that our inquiry is defective, rather than that the story is false. But evidence can aggregate even when inquiry is exploratory. A sufficient number and variety of attempts to fail to confirm the story can begin to provide evidence against it. Engaging in exploratory inquiry can thereby reap the epistemic benefits of truth-directed inquiry—it can yield truths that might otherwise be hidden—while minimizing the risks.

It must be emphasized that while exploratory inquiry minimizes the risks to knowledge, it doesn’t eliminate them. There’s no way to ensure our inquiry is genuinely exploratory: that we’re really placing sufficient weight on the official story. The very fact that there’s no principled method, let alone an algorithm, to assess the justified weight that ought to be placed on the official story, relative to the results of lay inquiry, ensures that we can inadvertently slide from exploratory into truth-directed inquiry. That is, we can take ourselves to be placing an appropriate weight on the official story, but be overimpressed by our failure to confirm it. There’s room for reasonable disagreement here: there are cases where we might reasonably disagree over whether an agent is right to take her repeated failures to confirm the official story as evidence against it.^12^ It should be noted, too, that attempts to safeguard against this risk lower the (already low) probability of reaping epistemic benefits. By its nature, exploratory inquiry greatly reduces the probability of false negatives (seeming to disconfirm the official story) and thereby increases the probability of false positives (taking the story to hold up to examination).

In the absence of an algorithm or a method to ensure that we’re placing appropriate weight on the official story, relative to our own inquiries, how do we act on the advice ‘engage in exploratory, not truth-directed inquiry’? While there’s no foolproof way to proceed, I suggest we engage *dogmatically*. When Kripke (2011) advised dogmatism, he had in mind refusing to engage with an argument we know to be misleading. Such dogmatism is appropriate for most of us, most the time when it comes to official stories: we lack the time, tools and motivation to investigate them (in any serious way), so we appropriately ignore challenges to them (Levy, 2021a, b). But in addition to dogmatic refusal to engage, there’s also dogmatic engagement. When we engage dogmatically, in the manner I advocate, we *expect* to be unable to counter the arguments we’re presented with; we expect our inquiries to fail to confirm the official story (or disconfirm rivals to it). Our inquiry is dogmatic, because we don’t take prima facie good evidence against the story to be a reason to reduce confidence in it. To put it another way, we expect to encounter evidence that is misleading, in ways that we cannot identify. To engage dogmatically is somewhat akin to looking through a book of visual illusions: we expect to encounter what seems like strong evidence for things being thus-and-so without raising our credence that things are thus-and-so.^13^

All going well, engaging dogmatically in exploratory inquiry allows us better to understand the official story (and its rivals). Insofar as the strength of the official story is itself due to its response to challenges, such inquiry may even be indispensable if the layperson is to come to understand it. All going well, such inquiry leads to the grasping of connections, of causes, the capacity to answer *why* questions and to assess counterfactuals; the elements or markers of understanding. Of course, lay inquiry rarely goes all *that* well. Any serious investigation of an abstruse theory may lie beyond my capacities. Even in cases like that, however, my dogmatic engagement may yield *some* understanding; say, some sense of what the issues are. So long as my inquiry is sufficiently dogmatic—sufficiently exploratory—I stand to gain some understanding while retaining knowledge.

There’s a risk that dogmatic inquiry may inappropriately slide into truth-directed inquiry. I may take the anomalies I detect and my repeated failures to confirm the official story as evidence against it, when it is much better seen as evidence of my limitations and the defects of my inquiry. This risk can’t be entirely avoided: not without sacrificing the epistemic benefits of exploratory inquiry. Anomalies can aggregate, and an agent *can* appropriately take her failure to confirm the official story as evidence against it. She should recognize that the fault is more likely hers or her inquiry’s than the story’s; nevertheless there comes a point when she reasonably takes her concerns as a reason to worry; perhaps to seek confirmation or disconfirmation from people with different biases and different skill sets. Dogmatic inquiry is often the way to understanding. Sometimes, it may be a way to new truths. It thereby benefits the agents who undertake it, and the epistemic community as a whole.

## Conclusion

The call to do our own research is seductive. Autonomy is a central value for many of us, and few want to be seen as mere followers of the herd. In this paper, I’ve argued that we ought to be wary of doing our own research. It’s no accident that contrarian theorists especially laud it: their independent research (or the independent research of those in their circles) has indeed been crucial in leading them to their views. Doing their own research has cut them free from the moorings of truth. When there’s an expert consensus, or expertise is required for a reasonable view on a topic, doing our own research risks truth and justification.

But doing one’s own research can have epistemic benefits. Cases in which laypeople are able to correct the experts are unusual, but they do occur, especially when the expert consensus represents too narrow a range of viewpoints. Correction may be rare, but when it occurs it may also be very significant. Moreover, there are other benefits to lay research, such as the capacity to apply political pressure when needed. Finally, doing one’s own research tends to increase understanding, even when it undermines knowledge. These facts entail that the attractions of doing one’s research are genuine.

I suggested that we can garner most (though not all) the benefits of doing one’s own research while greatly reducing (though not eliminating) its risks by engaging in what I have called exploratory, rather than truth-directed, research. The agent engaged in exploratory research *expects* to have great difficulty in confirming the official story (or, for that matter, in disconfirming rival accounts). Because she expects these results, however, she is prepared also to discount them: to see them as providing much better evidence for the defectiveness of her inquiry than for the falsity of the official story. By engaging dogmatically, she comes to understand the official story and its rivals, while minimizing the risks to truth. At the same time, she remains prepared to place *some* weight on a sufficient number and diversity of failures, especially when no special equipment or intellectual tools is needed to test the theory. Typically, the dogmatic inquirer won’t move even then to real doubts about the story. She’ll consult with others first, ideally a range of others, and especially those with more expertise than her.

None of this is foolproof. Far from it. We can easily deceive ourselves, thinking we are engaging dogmatically while placing too high a weight on the results of our own inquiry. We can also place too much weight on the official story, though I think cases like this will be much rarer. We are limited epistemic beings, and foolproof methods of inquiry are not available for us. If I’m right, though, dogmatic or exploratory inquiry offers us our best hope for balancing the risks and rewards of doing our own research.

Finally, it’s important to confront the worries I earlier deferred. How do we know that a topic falls within the purview of an epistemic authority and—more pointedly—how do we know that epistemic authorities are reliable? There are many historical cases in which apparent authorities have promulgated false theories, often knowingly so. Soviet scientific institutions were as advanced as any in the world, but they advocated Lysenkoism for purely political reasons (and with disastrous consequences). Such cases are by no means confined to totalitarian states: it’s all too easy to identify (albeit smaller) scientific scandals in recent democracies like the United States. Just one example can stand in for many: partisan political forces succeeded in distorting public discussions of acid rain sufficiently to cause a consumer of science as sophisticated as Naomi Oreskes to believe in the early 1990s that there was ongoing scientific controversy over its causes (Oreskes & Conway, 2011). Given that science can be corrupted, or led astray by ideology or money, how can we be confident that deference is reliable?^14^

I have already blunted some of the force of this worry by clarifying what I mean when I say that the expected epistemic return on deference is greater than that of self-reliance. Self-reliance is so unreliable that it rarely performs better than even unreliable epistemic authorities. It is nevertheless important to address worries concerning our capacity to identify the reliable epistemic authorities we may defer to in good conscience. This is a difficult and specialized task, and one on which individual cognition is no more reliable than on other difficult and specialized tasks. We can no more identify genuine epistemic authorities on our own than we can answer important scientific questions on our own. We are reliant on others—on those very institutions, and the institutions of civil society they inform—to identify them for us as the authorities to defer to, and we rely on the scientific community to keep them (that is, themselves) honest. To that extent, the proposal that we ought to defer to epistemic authorities begs the question against those who doubt that the authorities are reliable. I claim we ought to defer to those institutions who identify themselves as the institutions to defer to, when (and only when) the other institutions of civil society accept this claim. If, as in Soviet Russian (or for a brief time in the United States, on the much more local issue of acid rain) both institutions and civil society misfire or are corrupted, we will unknowingly defer to unreliable authorities.

We would much prefer to have an institution-independent guide to epistemic authority that we could use in the absence of genuine expertise on a topic. But we can no more have that than we can have the ability to adjudicate on the first-order claims within the purview of the epistemic authorities. To some extent, the overall reliability of the epistemic authorities is attested by the functioning of the societies in which they’re important institutions. Soviet agriculture, and thereby the whole nation (indeed, much of the Eastern bloc) suffered disastrous consequences from the promotion of Lysenkoism. But for multiple reasons, such functioning is weak and unreliable evidence for the reliability of epistemic authorities: problems in such authorities will not show up in society for some time; the linkage between their reliability and good functioning is weak and indirect; problems in some specific domain might be severe and yet compensated for by other factors, and so on. In the end, there is nothing to guarantee that the epistemic authorities are reliable that is genuinely external to them and to the (almost equally opaque) institutions of civil society itself.

We can’t have any guarantees. The epistemic condition, for beings like us who are pervasively dependent on distributed cognition and specialized inquiry, is to rely on institutions and individuals to perform their tasks sufficiently well for reliability. We have to trust them: not in their good will (some will certainly fail to exhibit it), but for the proper functioning of the multiple mechanisms that promote error correction, the cancelling out of bias and the eventual identification of corruption. The history of science seems to indicate that for the most part, however, these mechanisms work sufficiently well most of the time. So the experts tell me, and I believe them.^15^

## Footnotes

1 Of course, this brutally compressed glimpse of the history of philosophy presents a cartoon version of the actual thought of these philosophers. Kant, for example, had a more complex and subtle view of the epistemic import of testimony than one would glean from his famous essay on the Enlightenment. See Gelfert (2006) for a useful corrective on his views.

2 On what is perhaps the best-known account of intellectual autonomy, an agent is autonomous to the extent she has been active in resolving conflicts between her beliefs (Zagzebski, 2013). Rival accounts often take their cue from work on moral autonomy. Elzinga (2019) models his account on relational views of moral autonomy, arguing that intellectual autonomy requires a sense of oneself as a full member of the epistemic community, while Carter (2021,2022) models his account on historical accounts of moral responsibility, and requires that the agent possesses a history in which her cognitive faculties have not been bypassed. On these accounts, intellectual autonomy is fully consistent with deference to testimony. Dissenters from these views are rare. Fricker (2006) is one of the few who continues to equate intellectual autonomy with self-reliance. Depending on how her account is best interpreted, Tanesini (2021) might also be a dissenter. On her account, an agent must have her own reasons for her beliefs to count as autonomous. She maintains that someone can be in autonomous in beliefs acquired through testimony “if she is capable of evaluating her belief based on testimony even though she chooses on that occasion merely to trust another person.” It’s unclear what the ‘evaluation’ she appeals to here consists in; if it requires grappling with the first-order evidence oneself, then she counts as a dissenter.

3 Ballantyne and Dunning (2022) is a rare challenge to the near-consensus. They caution against doing one’s own research on the basis of research indicating that novices quickly become overconfident in their own judgments (e.g., Sanchez & Dunning, 2018). I take the problem to be much more general than that: it’s not only near-novices who are at risk of losing knowledge from doing their own research; so is everyone who is not an expert in the narrow domain of inquiry.

4 Ballantyne (2019) describes incursions of an expert into a field adjacent to her own as *epistemic trespassing.* He largely agrees that the risks of such trespassing are high, but also provides examples he takes to involve successful transfer of skills across domains. However, his examples involve relatively simple tasks in an expert domain, and the bar for success was set low; genuine experts still outperformed those who lacked field-specific expertise by a very large margin.

5 At the suggestion of a reviewer for this journal, I’ve weakened this claim slightly: I initially wrote that deference to the consensus was a reliable way of acquiring knowledge. The reviewer rightly points to the recent replication crisis in a number of sciences: given the extent of the problems the crisis has revealed, we should conclude that expert confidence in these fields until recently reflected genuine knowledge much less often than we’d like. While the situation has since improved (both insofar as the questionable research practices that very significantly gave rise to the replication crisis are less widespread today, and insofar as many unreliable consensuses have now collapsed), the crisis is ongoing and it is reasonable to expect that some findings that today seem reliable will fall to it. Moreover, as the reviewer also points out, the narrowness of genuine expertise raises the worry that a consensus is not well-founded, because experts are talking past one another: indeed, Millgram (2015) thinks specialization often gives rise to this kind of concern.

6 It’s worth noting a caveat, urged by a reviewer for this journal. The literature on inductive risk (building on Douglas, 2000) emphasises the extent to which all factual enquiries are value-laden: at minimum, researchers must make decisions about how to weigh the risks of false positives against those of false negatives, and these decisions reflect their values. Elsewhere, I’ve suggested that this gives us a reason to prefer the testimony of experts who seem to share our values to experts with different values (Levy, 2019), but ascertaining the values embedded in scientific findings is difficult. The reviewer suggests that knowing about inductive risk should make us less deferential. It is surely correct that it gives us a reason to be less confident in scientific findings, but it remains the case that we are unlikely to do better by doing our own research; at best, we will tend to do no worse.

7 There is more than one *kind* of understanding, of course. Propositional understanding consists in understanding *that* something is the case. Here, I’m concerned with what is sometimes called explanatory understanding: understanding *why* something is the way it is.

8 Of course, the contrarian thinker does tend to acquire knowledge through her own research. She may now know things like *the melting point of steel*, for instance. Insofar as our own research leads us to acquire beliefs that accord with the official consensus, we’re not risking knowledge. Since understanding depends on knowledge, the contrarian may even come to understand *proximate* causes. Suppose she comes to believe that the collapse was caused by the impact of the planes followed by the heat of the fires weakening its internal structure, but thinks that the Saudis were dupes of the CIA. In that case, she has a true belief about its proximate causes and may be sufficiently well versed in the causal mechanisms to count as understanding the collapse. But she has a false belief about its distal causes and cannot be said to understand why the World Trade Center collapsed, when the question has a broad scope.

9 There’s empirical evidence to back up these claims: Dan Kahan has shown that those on the political right who believe that climate change is not a significant problem tend to know *more* about the mechanisms involved than those who accept it is a significant problem (Kahan, 2015). They may manifest more understanding of climate change than many people who know that climate change is a significant problem.

10 The distinction between exploratory and truth-directed research is orthogonal to the distinction between shallow and deep research. Shallow research is research aimed at finding out what well-credentialed sources say about an issue and consists in consulting resources aimed at laypeople, while deep research is research that engages with the first-order evidence. Exploratory research is research that aims at understanding, while truth-directed research aims at truth. Those who advocate doing one’s own research have in mind an ideal of deep, truth-directed research, but research can be shallow and truth-directed or deep and exploratory.

11 That’s not to say that in principle we cannot encounter evidence that counts against a theory of this sort. Rather, the claim is that we cannot encounter evidence that counts against a theory of this sort *by testing the theory* (where ‘testing the theory’ includes trying to confirm rivals to it). Other kinds of evidence could count against the theory: e.g. documentary evidence that the world’s scientists are involved in a conspiracy to fabricate evidence.

12 It might be worth highlighting the parallel between exploratory inquiry and what Kuhn (1970) calls normal science. Inquiry in normal science is truth-directed, of course, but the scientist’s attitude to anomalies—results that conflict with the reigning paradigm—is akin to the explorer’s attitude to her findings. She does not see such anomalies as a reason to abandon the paradigm. But like the explorer, scientists will take the challenge to a paradigm seriously when the accumulation of anomalies crossed some ill-defined, and perhaps undefinable, threshold.

13 For a number of reasons, ranging from justifiable demands of civility through to psychological facts about social animals like ourselves, it can be difficult and often inappropriate to engage dogmatically in person with someone who is sincere in their views. For this reason, dogmatic engagement might be better confined to engagement with texts rather than persons.

14 A reviewer for this journal points to an additional concern: there is extensive evidence that expertise in some domains does not correlate with a greater capacity to make predictions about future events in that domain or even to bring about the outcomes the field aims at. For instance, Tetlock (2005) found that economic and political predictions by supposed experts were no more reliable than those of non-experts, and Dawes (1996) found that outcomes for the patients of psychiatrists and psychologists were no better than those of the patients of minimally trained counsellors. As the reviewer notes, these findings don’t indicate that there is no such thing as expertise: rather, they indicate there are domains where it fails at certain tasks that seem within its purview. The reviewer suggests that this gives us reason to defer to experts in what Kahneman and Klein (2009) call *high-validity domains*; domains in which expertise is shaped by fast and reliable feedback. The reviewer suggests, that is, that instead or as well as choosing to trust experts by reference to the criteria philosophers like Goldman (2001) have suggested, we attend to the domain of expertise. Elsewhere (Levy, 2022), I’ve expressed scepticism about the capacity of laypeople to assess putative experts by reference to Goldman-style criteria, on the grounds that these criteria are easily mimicked. Domain is much harder to mimic, and ordinary people can readily learn that expert judgments in some domains should not be regarded with much credibility (though it remains likely that even in these domains we do no worse by deferring than by relying on our own judgment).

15 I am grateful to two reviewers for this journal for very helpful comments. I am also grateful to the audience at the 2021 Australasian Philosophical Association, where an earlier version of this conference was delivered as the Presidential Address. Work leading up to this paper was supported by a generous grant from the Australian Research Council (DP180102384).
